# Optimisation of Intervertebral Disc Mechanical Properties and the Impact of Vertebral Alignment in Subject‐Specific Finite Element Models

**DOI:** 10.1002/cnm.70052

**Published:** 2025-06-02

**Authors:** Emily S. Kelly, Akbar A. Javadi, Timothy P. Holsgrove, Michael Ward, David Williams, Jenny Williams, Cathy Holt, Judith R. Meakin

**Affiliations:** ^1^ University of Exeter Exeter UK; ^2^ Cardiff University Cardiff UK

**Keywords:** finite element modelling, intervertebral disc properties, lumbar spine, subject‐specific modelling

## Abstract

Subject‐specific finite element models could improve understanding of how spinal loading varies between people, based on differences in morphology and tissue properties. However, determining accurate subject‐specific intervertebral disc (IVD) properties can be difficult due to the spine's complex behaviour, in six degrees of freedom. Previous studies optimising IVD properties have utilised axial compression alone or range of motion data in three axes. This study aimed to optimise IVD properties using 6‐axis force‐moment data, and compare the resultant model's accuracy against a model optimised using IVD pressure data. Additionally, model vertebral alignment was assessed to determine if differences between imaged specimen alignment and in vitro 6‐axis test alignment affected the optimisation process. A finite element model of a porcine lumbar motion segment was developed, with generic IVD properties. The model loading and boundary conditions replicated in vitro 6‐axis stiffness matrix testing of the same specimen. The model was then optimised twice, once using experimental IVD pressures and once using forces and moments. A second model with geometry based on the specimen's vertebral alignment from the 6‐axis testing was also developed and optimised. The 6‐axis force‐moment optimised model had more accurate overall 6‐axis load‐displacement behaviour, but less accurate IVD pressures than the pressure optimised model. Neither optimised model fully captured spinal behaviours, due to model and optimisation process limitations. The 6‐axis vertebral alignment model had lower error and different optimised IVD properties than the imaged vertebral alignment model. Thus, vertebral alignment affected segment stiffness, so should be considered when developing spine models.

## Graphical abstract

Optimisation methods were compared for a porcine lumbar intervertebral disc model. Neither optimising with 6‐axis forces and moments nor optimising with disc pressures fully captured spinal behaviour. Addressing model limitations could improve the process. Flexion‐extension optimisation was more accurate with geometry based on experimental rather than imaged vertebral alignment.

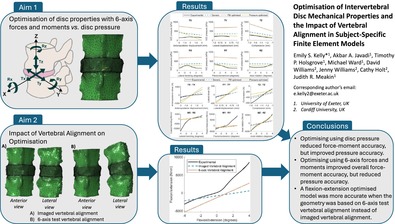

## 1 Introduction

Low back pain is a major contributor to disability worldwide and is increasingly prevalent [1]. There are many known causes and risk factors for low back pain; however, a significant proportion of people experience non‐specific or unexplained pain [1, 2]. An improved understanding of spinal biomechanics and loading, and how they may vary between people, could aid in the diagnosis and treatment of back pain. However, in vivo spinal loading is challenging to assess, and so in silico methods are often utilised to provide a greater level of detail than can feasibly be achieved in vivo. For example, finite element modelling has contributed greatly to knowledge in areas such as scoliosis and vertebral fractures [3, 4]. Modelling studies have also helped understand intervertebral disc (IVD) functionality and degeneration, considering both biomechanical and biochemical aspects [5, 6].

Subject‐specific finite element models can be used to assess differences in spinal loading between individuals. Many spine models incorporate subject‐specific geometries from imaging data [7, 8, 9, 10], and subject‐specific motion can be incorporated into displacement‐controlled models using kinematic data from radiographs or fluoroscopy [10, 11, 12]. However, the IVD is often modelled with generic properties, with few studies accounting for the variations in IVD properties between people that may result from structural differences or disc degeneration. Those that have varied IVD properties show an impact on spinal loading [9], suggesting that the inclusion of subject‐specific IVD properties could improve the accuracy of computational model predictions.

One method to incorporate subject‐specific IVD properties is to optimise generic properties based on experimental data. Mengoni et al. [13, 14] have shown that bovine IVD behaviour under compression can be calibrated by optimising annulus fibrosus fibre stiffnesses. Gruber et al. [15] showed that an IVD model can be optimised using generic flexion, extension, lateral bending and axial rotation range of motion data from literature. Thus, optimisation processes can be effective for generating subject‐specific models. To build from these previous studies, direct comparisons to experimental data in all six spinal degrees of freedom would be beneficial.

Generally, optimisation or validation of lumbar spine models includes comparing intradiscal pressures and ranges of motion due to pure moments [7, 8, 16, 17]. Shear effects, which also occur in the spine, are rarely considered. Optimisation could be performed with load–displacement data from in vitro 6‐axis mechanical testing [18, 19]. This would allow modelled IVD behaviour to be tuned to all six degrees of freedom, including accounting for multi‐axis and asymmetric effects [18]. Thus, 6‐axis data may provide advantages over intradiscal pressure or pure moment data for direct calibration of IVD properties.

One of the difficulties in utilising 6‐axis data for optimising IVD properties may lie in the challenge of ensuring the loading coordinate system position is consistent between the model and experimental data. The position of an applied axial compressive load in a model has been shown to influence the predicted vertebral stiffness [20]. With loading in six axes, and interplay between the axes, the accuracy of the coordinate system position may be even more crucial. Additional complications arise from the flexibility of spinal motion segments. The model geometry, based on imaging data, may not correspond to the relative positions of the vertebrae within the 6‐axis test rig prior to the stiffness characterisation. It is unclear how much of an impact this discrepancy between in silico and in vitro vertebral alignments may have on the optimisation of IVD properties.

Thus, the purpose of this study was to investigate the use of 6‐axis forces and moments in optimising subject‐specific IVD behaviour, through the following objectives:

1. Compare the accuracy of optimising subject‐specific IVD mechanical properties using IVD pressures versus 6‐axis forces/moments;
2. Compare the optimisation accuracy and optimised IVD properties for model geometries based on imaged versus 6‐axis testing vertebral alignments.

## 2 Materials and Methods

### 2.1 Imaging and In Vitro 6‐Axis Mechanical Testing

A porcine lumbar spine specimen was obtained fresh from an abattoir within 2 h post‐mortem, and then frozen at −20°C whilst wrapped in saline‐soaked gauze and double‐bagged to prevent dehydration. The specimen was thawed prior to being imaged, and then underwent a second freeze–thaw cycle between being imaged and mechanically tested. Imaging was performed on the full lumbar section using both MR and CT scanners to capture IVD and bone geometries, respectively. The MR imaging was performed using a 3 T Magnetom Prisma scanner (Siemens, Germany). A T2 TSE sequence was used with repetition and echo times of 8290 and 65 ms, producing data with a resolution of 0.8 × 0.8 × 0.8 mm, and a gap between slices of 0.08 mm. The CT imaging was performed using a Biograph Vision 600 (Siemens, Germany) and reconstructed with a resolution of 0.5 × 0.5 × 0.5 mm. Both sets of imaging were captured in the same session, with the specimen kept in a consistent position within a tray that was transported between scanners. Automatic registration and segmentation of the CT and MR images were performed using Mimics 26.0 (Materialise NV, Belgium). Automatic registration was performed using the software's built‐in tool and was visually assessed to confirm image alignment. Bone geometries were segmented from the CT images and combined with the IVD geometry, segmented as a single domain from the MR images. The segmented geometries were then exported to 3‐matic 18.0 (Materialise NV, Belgium) for meshing.

In preparation for the in vitro mechanical testing, the specimen was dissected to isolate the L1‐L2 level. All soft tissues except for the IVD and ligaments were removed, and parallel cuts were made using a bone saw to provide flat inferior and superior vertebral body surfaces. The cut surfaces were defined using a 3D printed guide that had been modelled within the 3‐matic software to help ensure they were consistent between the physical specimen and the finite element model. Finally, the posterior elements were removed so that the specimen consisted of the vertebral bodies and interjacent IVD, with the anterior and posterior longitudinal ligaments intact. Removal of the posterior elements eliminated potential sources of error between the experimental and modelling data from facet joint interactions, ensuring characterising the IVD behaviour was the study's focus. The longitudinal ligaments were left intact to prevent inadvertent damage to the IVD from removing the ligaments.

The specimen was potted using dental acrylic (Pegasus Pourable Cold Cure Denture Base, Schottlander, UK), with k‐wires used to locate the specimen within the pot, so that the alignment of the load cell with the specimen was known. A fibre optic pressure transducer (FISO LS catheter 2Fr, 0–20 bar range) was inserted, via a 16G non‐coring needle and Tuohy‐Borst adapter, with an anterolateral approach to record pressures at the centre of the IVD. The transducer had an accuracy of ±20 kPa (1% of the full range) and a resolution of ±0.3 mmHg.

**FIGURE 1:**
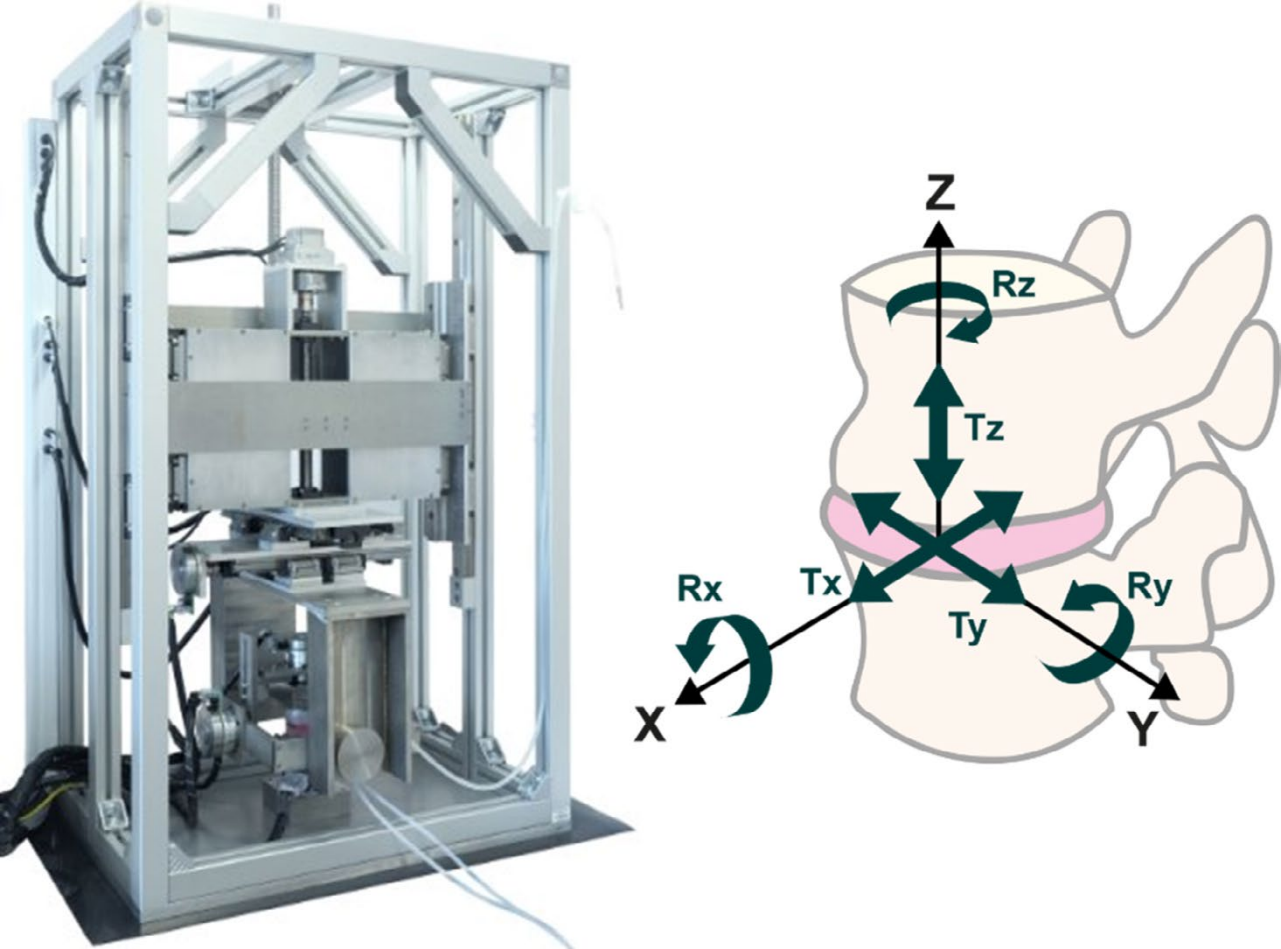
6‐axis mechanical test rig, displaying the six translations and rotations applied to the specimen during the stiffness matrix tests. Figure adapted from [21] under a CC BY 4.0 license.

Detailed methods for the 6‐axis stiffness matrix tests have been previously reported [18], and are summarised here. The lower vertebra was fixed in place, and the upper vertebra was allowed to settle into a neutral position, where the force and moment outputs were zero. An axial compressive preload was then applied and held for 30 min to allow the specimen to reach a loaded equilibrium, resulting in a compressive displacement of 1.4 mm. Each axis was then tested in turn, in position control, applying ±3 mm anteroposterior translation (TX), ±1.5 mm lateral translation (TY), ±0.4 mm axial displacement (TZ), and ±4° lateral bending (RX), flexion‐extension (RY) and axial rotation (RZ) (Figure 1). The ranges of motion and use of position control have been previously established in 6‐axis stiffness testing as representative of typical physiological motion [18, 22]. The translations and rotations were applied around a point at the centre of the disc, with a travel distance error of 0.025 mm and transmission accuracy of 0.025°. The centre of the disc was defined in 3‐matic using disc height, anteroposterior and lateral measurements. This central point was translated to the physical testing rig through the use of the k‐wire guides locating the specimen within the pot, and input parameters in the experimental control model setting the distances between the load cell, disc centre and superior vertebral body cut surface.

For each axis test, the other five axes were kept stationary, and five sine wave cycles of loading were applied at 0.1 Hz. The final three cycles were averaged for use in the model optimisation. IVD pressure and force and moment outputs for each of the six degrees of freedom were measured during the tests at a frequency of 100 Hz. Previous tests have demonstrated that the errors between desired and actual loads in 6‐axis load control tests were within two times the noise floor of the load cell at 0.55 N (anteroposterior force), 0.53 N (lateral force), 1.62 N (axial compression) and 0.01 Nm in all rotational axes [21].

### 2.2 Finite Element Model Development

The model mesh was generated in 3‐matic, before being exported to Abaqus 2022 (Dassault Systèmes, France). A quadratic tetrahedral mesh was used, with maximum element sizes of 2.5 mm in the bone and 0.825 mm in the IVD. Mesh convergence was assessed on a previous specimen model, with the chosen mesh producing disc stress components within 2.5% of the finest mesh under typical ranges of motion in flexion, extension, lateral bending and axial rotation.

The IVD was modelled with linear elastic orthotropic material properties and a cylindrical coordinate system originating at the centre of the IVD. For the generic model, the compressive modulus (E3) was estimated from the compressive in vitro data, and generic values were used for the remaining elastic and shear moduli and Poisson's ratios (Table 1). The anterior and posterior longitudinal ligaments were implemented as 2D axial spring connectors, acting in tension only. The ligaments were assigned nonlinear elastic stiffness properties adapted from literature [16], implemented as force‐displacement curves accounting for ligament length in the present model. The bone was modelled as isotropic and linear elastic with an elastic modulus of 3500 MPa and Poisson's ratio of 0.25, as a balance of cortical and trabecular properties [9, 16, 23].

**TABLE 1:** Generic orthotropic IVD material properties.

Engineering constants	
E1, E2 (MPa)	1
E3 (MPa)	0.75
ν12, ν13, ν23	0.425
G12, G13, G23 (MPa)	0.5

**FIGURE 2:**
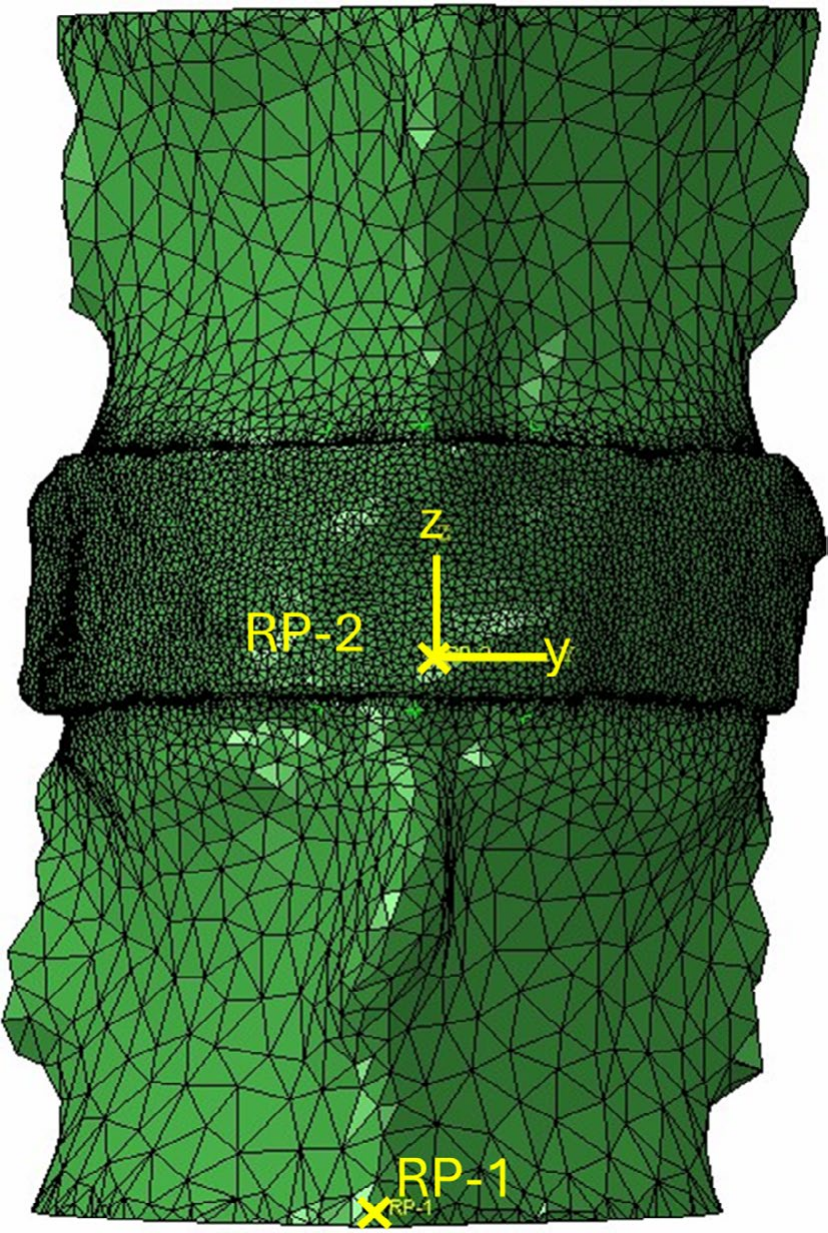
Finite element model of the L1‐L2 porcine specimen, showing the inferior vertebra fixed constraint reference point (RP‐1), the superior vertebra loading reference point (RP‐2) and the loading coordinate system.

Loading and boundary conditions were applied to replicate the in vitro 6‐axis tests. The model was displacement‐controlled, with kinematic couplings for each of the vertebral bodies tying their nodes to translations and rotations applied at reference points. The inferior vertebra was fully constrained in translation and rotation via a reference point (RP‐1) at the centre of the inferior vertebral body cut plane. Kinematics were applied to the upper vertebra via a reference point (RP‐2) that reflected the centre of rotation and local loading coordinate system from the mechanical testing (Figure 2). The 1.4 mm axial compressive preload was applied, followed by displacements and rotations in each of the six axes, with the other five axes constrained from moving. Model predictions for the 6‐axis forces and moments were obtained, using the same reference point (RP‐2) and coordinate system as was used to apply the kinematics. Pressure predictions, defined as the equivalent pressure stress, were also obtained at the centre of the IVD to correspond to the experimental measurements.

### 2.3 IVD Material Property Optimisation

All optimisation processes were carried out in Isight 2022 (Dassault Systèmes, France), on an 8‐core Intel Core i7‐10700 computer with 32 GB RAM. For each process, the nine orthotropic engineering constants representing the model IVD properties were optimised, based on specified allowable ranges (Table 2). A narrower range was given for the compressive stiffness (E3), due to the more accurate estimation of the material property from the in vitro data.

**TABLE 2:** Allowable ranges for the IVD properties used by the optimisation process.

IVD property ranges	
E1, E2 (MPa)	0.05–8
E3 (MPa)	0.6–2.4
ν12, ν13, ν23	0.375–0.499
G12, G13, G23 (MPa)	0.01–6

To compare optimising using IVD pressures versus 6‐axis forces and moments, the optimisation process was performed twice using the non‐dominated sorting genetic algorithm, NSGA‐II. NSGA‐II is multi‐objective and selects the best solutions from each generation to carry forward to the next [24]. A population size of 12 was used, with 20 generations and a crossover probability of 0.9. The values were adapted from previous literature [15].

The IVD properties were optimised by minimising normalised mean square errors (NMSE) between the experimental and model outputs. In each case, the error was normalised by dividing by the standard deviation to account for the differences in magnitude between the forces and moments being optimised by the same process. For the force‐moment optimisation, 36 error values were minimised, accounting for the six force and moment outputs (FX, FY, FZ, MX, MY, MZ) for each of the six input kinematics (TX, TY, TZ, RX, RY, RZ). The errors in the principal axes, such as the anteroposterior force caused by an anteroposterior displacement (FX‐TX), were given double weighting. The pressure optimisation had one IVD pressure error per input kinematic, resulting in six total error values. Each optimisation process was set to terminate when all error values were below a threshold of 0.1 or when the iteration limit was reached, as governed by the number of generations.

### 2.4 Comparison of Vertebral Alignments

To investigate the impact of vertebral alignment on the model predictions and optimisation process, a second model was developed that reflected the 6‐axis testing initial vertebral alignment. During the 6‐axis testing, the vertebral alignment changes as the specimen is placed in the test rig and the superior vertebra is allowed to settle into an unloaded neutral position. Additionally, as the compressive preload is applied, the specimen may move in the other five axis directions to maintain zero load conditions in the other axes. These translations and rotations were recorded, and corresponded to the difference in vertebral alignment between imaging and 6‐axis testing conditions. Translations in the TZ axis (axial compression) were considered part of the preload rather than the vertebral alignment, due to the compressive forces generated.

**FIGURE 3:**
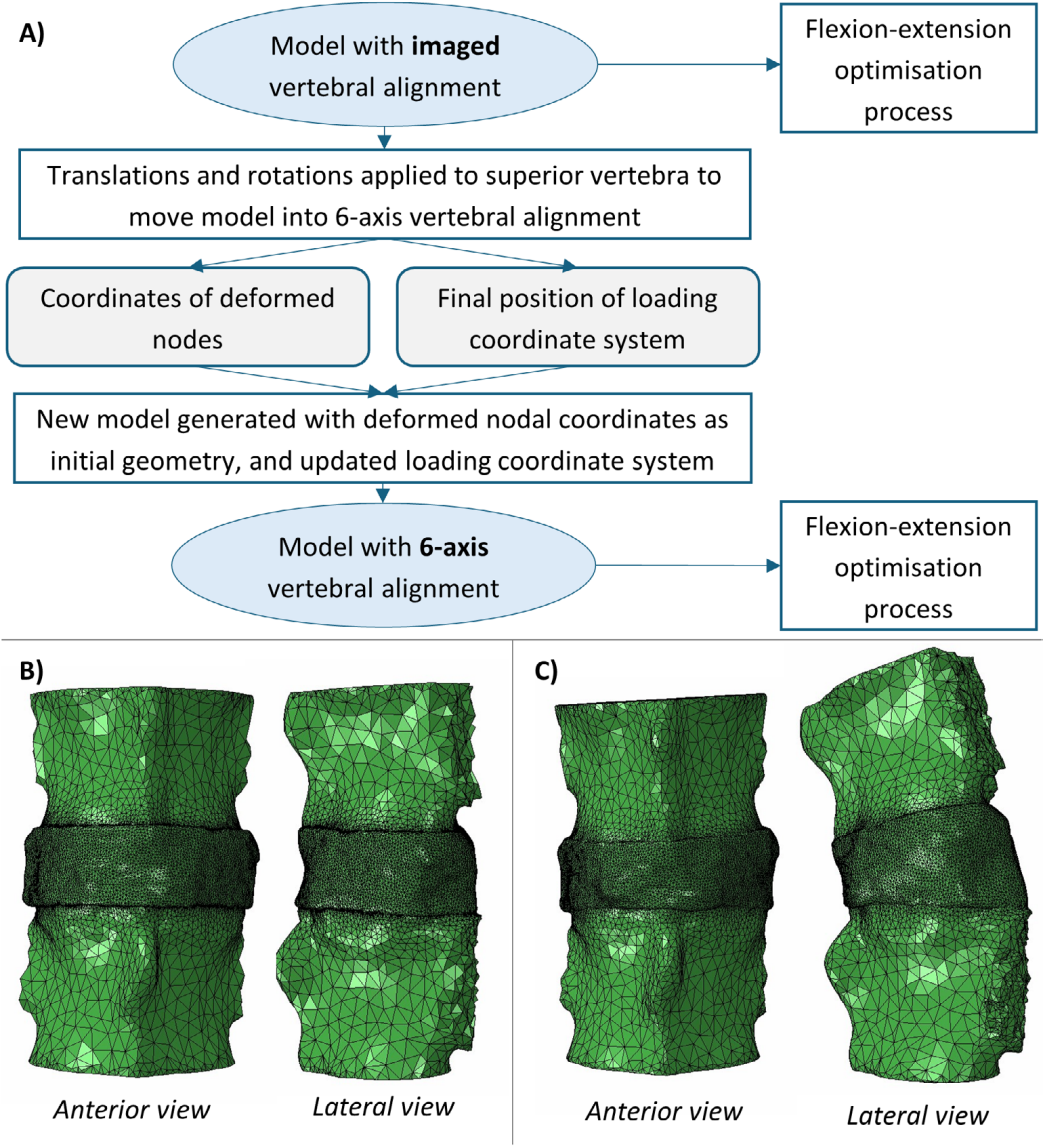
(A) Flowchart displaying the procedure to generate the 6‐axis vertebral alignment model from the imaged vertebral alignment model; (B) Original model with geometry based on imaged vertebral alignment; (C) Model with geometry based on 6‐axis test vertebral alignment.

The kinematics for the difference in vertebral alignment were applied to the original model, which had geometry based on the imaged vertebral alignment (Figure 3). This provided deformed nodal coordinates and an updated position of the loading coordinate system, which were representative of the 6‐axis test conditions. A second model was then generated, with a geometry that used the deformed nodal coordinates, and so had the vertebral alignment from the 6‐axis testing without generating undesired forces and moments from the alignment change. The rest of the model input parameters were assigned as previously described.

The original model with geometry based on the imaged vertebral alignment and the second model with geometry based on the 6‐axis testing vertebral alignment were then both optimised using flexion/extension data. By focussing on a single axis, the optimisation process was simplified to ensure convergence in a timely manner. Flexion/extension was chosen as it is an important motion for many daily activities, and may be impacted by differences in vertebral alignment. As this optimisation only had a single objective, minimising the NMSE in the flexion/extension principal axis (MY‐RY), a Downhill Simplex optimisation method was used. The method is a heuristic search where a simplex moves and contracts towards the minimum [25]. The initial simplex size governs the locality of the search and was set to 0.5 (i.e., 50% of the total design space) to avoid local minima. The initial IVD properties were the generic properties, and the same allowable ranges were utilised as for the previous optimisations (Table 2). The optimisation terminated when the error converged.

### 2.5 Analysis

The accuracy of optimising the IVD properties using IVD pressures versus using 6‐axis forces and moments was determined by comparing the NMSE values for the model forces, moments, and pressures after each optimisation process. The optimised model errors were also compared to the errors from the generic IVD material model to assess the effectiveness of the optimisation process. Similarly, the impact of vertebral alignment on the modelling and optimisation process was assessed by comparing the force, moment and IVD pressure NMSE values for the flexion/extension optimised models with geometries based on the imaging and 6‐axis testing vertebral alignments. The optimised orthotropic constants were also compared to determine whether the model vertebral alignment affected the optimal IVD material properties determined.

## 3 Results

### 3.1 Optimisation Process Convergence

**FIGURE 4:**
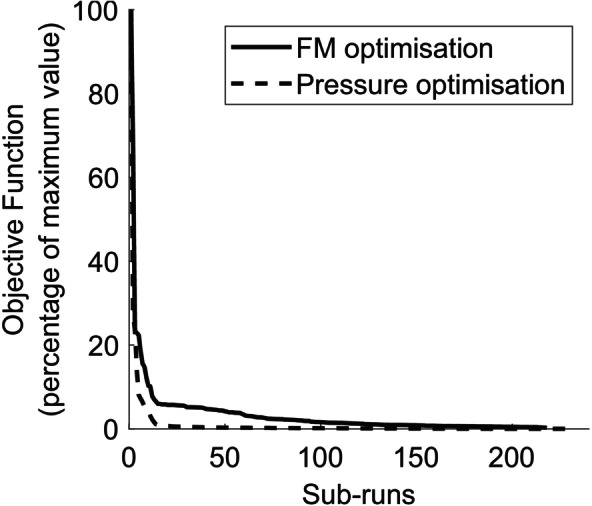
Objective function convergence for the force‐moment (FM) and pressure optimisation processes. Objective function is expressed as a percentage of the maximum objective function value.

Both the force‐moment and pressure optimisation processes terminated on reaching the iteration limit, rather than meeting the specified error threshold. Despite that, the objective functions, consisting of the NMSE values for each axis with double weightings for principal axes, appeared to converge (Figure 4).

### 3.2 Comparison of IVD Pressure and 6‐Axis Force‐Moment Optimisations

**FIGURE 5:**
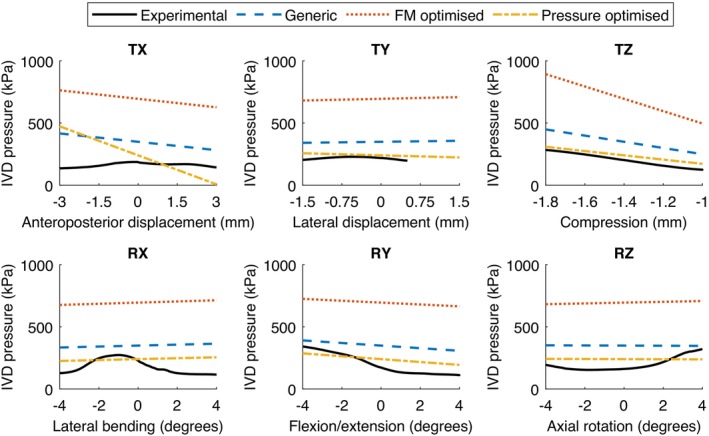
Comparison of IVD pressures from 6‐axis experiments and models with generic, force‐moment (FM) optimised, and pressure optimised IVD properties. All displayed data includes a −1.4 mm axial compression preload.

**FIGURE 6:**
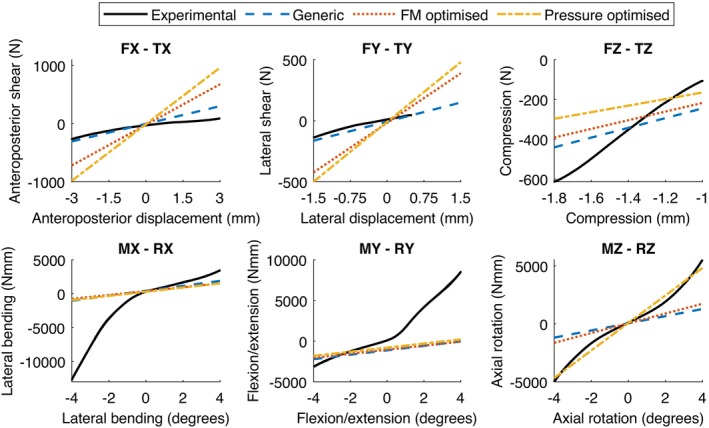
Comparison of principal axis load–displacement behaviour from 6‐axis experiments and models with generic, force‐moment (FM) optimised, and pressure optimised IVD properties. All displayed data includes a −1.4 mm axial compression preload.

The IVD pressure and principal axis force‐moment behaviours for the generic and optimised models were compared against the experimental data. Regarding the IVD pressure magnitudes (Figure 5), the pressure optimised model more closely reflected the experimental data than the generic or force‐moment optimised models, which both overestimated the pressures. Comparing the principal axes of the force‐moment behaviour (Figure 6), the generic model captured the experimental stiffness well in anteroposterior (FX) and lateral (FY) axes, with the optimised models overestimating the stiffnesses. In axial compression (FZ), all models underestimated the experimental stiffness. In lateral bending (MX) and flexion‐extension (MY), there was minimal difference between models compared to the degree to which the model behaviour underestimated the experimental stiffness. Finally, in axial rotation (MZ), the pressure optimised model accurately captured the experimental stiffness, whereas the generic and force‐moment optimised models underestimated the stiffness. Note that an error during the experimental data collection in the TY axis resulted in output measurements being cut off at +0.5 mm lateral displacement instead of achieving the full +1.5 mm displacement. This was accounted for in the error calculations, and should not have affected the overall trends observed.

The NMSE values from models with generic, force‐moment and pressure optimised IVD properties were compared for each of the 6‐axis input kinematics (Table 3), with lower NMSE indicating the model more accurately represented the experimental data. The IVD pressure optimisation led to a total pressure error change of −2760 compared to the generic model, with consistent pressure error reductions across all input kinematics. However, optimising using IVD pressure increased the error in the force‐moment model predictions by a total of +16,900 across all axes. The force‐moment optimisation led to a −1940 error change across the force‐moment axes and a +40,000 error change in the IVD pressure predictions. All error values are reported to three significant figures. The majority of the force‐moment error reductions occurred in the moment axes, for example, the axial rotation moment caused by anteroposterior translation (MZ‐TX) and all moments caused by axial compression (TZ) and axial rotation (RZ). Meanwhile, the force errors generally increased.

**TABLE 3:** Normalised mean square errors (NMSE) between model predictions and experimental data for forces, moments, and pressures for each input axis motion, showing the difference in NMSE for the force‐moment optimised (FM op.) and pressure optimised (pressure op.) IVD property models compared to the generic property model.

Input motion	Model IVD properties	NMSE for model outputs (*difference from generic model*)													
		FX		FY		FZ		MX		MY		MZ		Pressure	
TX	Generic	79.7		16.0		96.3		2240		5110		2970		1810	
	FM op.	922	*(+842)*	67.9	*(+51.9)*	99.2	*(+2.92)*	2030	*(−211)*	7520	*(+2410)*	1910	*(−1060)*	13,700	*(+11,900)*
	Pressure op.	2130	*(+2060)*	22.7	*(+6.65)*	257	*(+161)*	1360	*(−881)*	8680	*(+3570)*	35,500	*(+32,500)*	1500	*(−313)*
TY	Generic	7.56		6.51		12.3		2060		5470		1180		1320	
	FM op.	89.3	*(+81.7)*	328	*(+321)*	75.2	*(+62.9)*	2680	*(+624)*	5000	*(−478)*	1860	*(+676)*	16,500	*(+15,200)*
	Pressure op.	22.2	*(+14.6)*	519	*(+513)*	602	*(+590)*	3170	*(+1120)*	3160	*(−2320)*	3850	*(+2670)*	89.5	*(−1230)*
TZ	Generic	1.06		39.3		59.8		3500		32,300		1.38		360	
	FM op.	139	*(+138)*	174	*(+135)*	81.3	*(+21.5)*	2750	*(−751)*	28,200	*(−4040)*	1.20	*(−0.18)*	4140	*(+3780)*
	Pressure op.	20.7	*(+19.6)*	62.3	*(+23.0)*	167	*(+107)*	1330	*(−2170)*	16,500	*(−15,800)*	54.3	*(+53.0)*	24.5	*(−335)*
RX	Generic	8.92		90.4		254		4490		5300		510		492	
	FM op.	49.6	*(+40.7)*	123	*(+32.3)*	273	*(+18.8)*	4740	*(+253)*	4870	*(−423)*	510	*(+0.16)*	4050	*(+3560)*
	Pressure op.	16.5	*(+7.59)*	153	*(+62.7)*	337	*(+83.6)*	4590	*(+105)*	3580	*(−1720)*	360	*(−150)*	115	*(−377)*
RY	Generic	101		110		136		200		3920		228		264	
	FM op.	152	*(+51.0)*	239	*(+129)*	174	*(+37.9)*	162	*(−38.2)*	3920	*(−3.21)*	221	*(−6.65)*	2590	*(+2320)*
	Pressure op.	184	*(+83.4)*	135	*(+25.6)*	317	*(+181)*	148	*(−52.7)*	3630	*(−290)*	136	*(−92.5)*	49.3	*(−215)*
RZ	Generic	10.6		70.5		90.3		855		4990		1760		369	
	FM op.	10.5	*(−0.09)*	119	*(+48.2)*	128	*(+37.8)*	834	*(−21.9)*	4440	*(−545)*	1380	*(−379)*	3620	*(+3250)*
	Pressure op.	18.6	*(+7.95)*	217	*(+146)*	281	*(+191)*	639	*(−216)*	2990	*(−2000)*	57.5	*(−1700)*	75.6	*(−294)*

*Note:* All values are reported to three significant figures, so differences in NMSE may not exactly correspond to the reported NMSE.

Abbreviations: FX/TX—force/translation in anteroposterior axis; FY/TY—force/translation in lateral axis; FZ/TZ—force/translation in axial compression; MX/RX—moment/rotation in lateral bending; MY/RY—moment/rotation in flexion‐extension; MZ/RZ—moment/rotation in axial rotation.

### 3.3 Impact of Vertebral Alignment

**FIGURE 7:**
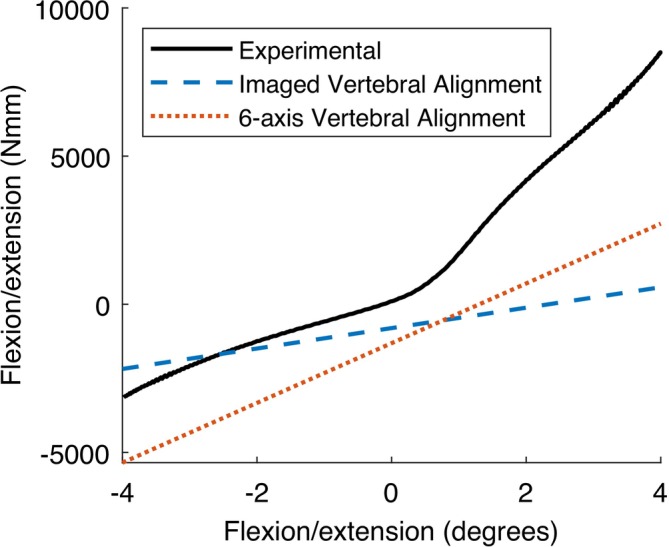
Comparison of flexion‐extension behaviour from 6‐axis in vitro experiments and models with geometry based on imaged vertebral alignment or 6‐axis vertebral alignment. Both models have flexion‐extension optimised IVD properties. All displayed data includes a −1.4 mm axial compression preload prior to applying flexion‐extension.

Following optimisation using flexion‐extension data, the model with geometry based on the 6‐axis vertebral alignment produced an NMSE in the flexion‐extension principal axis of 2266, which was lower than the error of 3308 in the original imaged vertebral alignment model. Using the 6‐axis vertebral alignment also resulted in similar model stiffness behaviour to the extension experimental data, though at an offset (Figure 7). There were large differences in the optimised IVD property values between the imaged vertebral alignment model and the 6‐axis vertebral alignment model (Table 4).

**TABLE 4:** Flexion‐extension optimised IVD material properties for models with geometry based on imaged vertebral alignment and based on 6‐axis vertebral alignment.

IVD property	Imaged vertebral alignment	6‐axis vertebral alignment
E1 (MPa)	7.4947	2.4923
E2 (MPa)	1.1527	0.68392
E3 (MPa)	0.61161	2.2957
ν12	0.408	0.42321
ν13	0.42413	0.39748
ν23	0.41725	0.38151
G12 (MPa)	0.65631	0.041292
G13 (MPa)	5.9937	1.2921
G23 (MPa)	5.9962	0.14564

## 4 Discussion

### 4.1 IVD Pressure and 6‐Axis Force‐Moment Optimisations

Optimising IVD mechanical properties using IVD pressure led to more accurate predictions for pressures, but generally did not produce accurate force‐moment behaviour in the spinal segment model. Meanwhile, optimising IVD properties using 6‐axis forces and moments reduced the overall error in 6‐axis load–displacement behaviour, but did not necessarily lead to improved behaviour in individual axes and also increased the IVD pressure error. Although both methods were partially successful, neither generated accurate subject‐specific IVD properties that fully captured IVD behaviour for this porcine model.

With regard to the pressure optimised IVD properties, despite the relatively accurate IVD pressure predictions, the model could not accurately reflect force‐moment behaviour, even in axial compression. Interestingly, the pressure optimised model did accurately capture the axial rotation principal axis behaviour (Figure 6). However, having only assessed a single specimen, it is unclear whether this was due to a relationship between IVD pressure and axial rotation or if it was coincidental. The model's inability to capture accurate force‐moment behaviour has implications for spinal model validation, as it shows that accurate model IVD pressures may not necessarily correspond to spinal behaviour as a whole. Note that this is specific to pressure comparisons utilising a single central pressure value to represent pressure in the IVD as a whole. IVD pressure distributions may correspond better to force‐moment behaviour, for example differentiating between flexion and extension [26]. However, in vivo and in vitro pressure measurements usually capture a single point of IVD pressure, so there is little way to validate or optimise a model using pressure distributions. IVD pressure may still be relevant for modelling, as one of the few metrics with in vivo measurements available for indirect validation [27]. However, the results of this study indicate that it should not be the sole component considered when validating or optimising IVD models, as ensuring accurate IVD pressure may not guarantee accurate forces and moments.

The force‐moment optimisation results highlight the challenges of using 6‐axis behaviour for direct optimisation of IVD properties. Optimisation of IVD properties using flexion‐extension, lateral bending and axial rotation can be successful. Gruber et al. developed an optimised model with an average *R* ^2^ value of 0.95 across the three axes when compared to literature range of motion data [15]. The limited effectiveness of the optimisation in the present study may be related to the addition of forces to the optimisation process. The moment error reductions coupled with force error increases suggest a potential trade‐off between optimising for accurate moments and for accurate forces. It is possible the optimisation tried to find a middle ground, leading to inaccuracies across all load–displacement behaviour.

As the optimisation terminated on reaching the iteration limit, it is possible the design space (consisting of the allowable ranges of each IVD orthotropic constant) had not been fully explored. Thus, there may have been other combinations of material property values that could have resulted in a lower error. The objective function did appear to converge (Figure 4). However, as the force‐moment optimisation took longer to converge than the pressure optimisation, there were still noticeable differences in error and material property values between the sub‐runs with the lowest error. The force‐moment optimisation took approximately 11 days to run based on the iteration limit that had been set, and so it was not feasible to increase that limit. However, the optimisation may have improved if allowed to run for longer, potentially aided by higher performance computing resources. Alternatively, the design space could be reduced by limiting the parameters optimised to only the most sensitive or narrowing the material property ranges, if the potential values could be estimated with higher accuracy.

A major limitation of the model was the use of linear elastic orthotropic material properties that represented the IVD as a single domain. In reality, IVD stiffness is anisotropic [18], with some individual axes also displaying non‐linear behaviour (Figure 6). This simplification of the IVD model may have resulted in the optimisation process being unable to identify a set of properties that could represent the force‐moment behaviour in all six axes. Both Mengoni et al. [13] and Gruber et al. [15] optimised IVD models with separate nucleus pulposus and annulus fibrosus regions, utilising material models that accounted for anisotropy, hyperelasticity and the annulus fibres. The simplified IVD model was used to reduce model complexity and thus the computation time of the optimisation processes. However, without these aspects, the effectiveness of the force‐moment optimisation may have been limited.

Similarly, the simplified IVD material model may have impacted the effectiveness of optimising using IVD pressure. While the optimisation achieved accurate IVD pressure results, the inaccuracy of the force‐moment predictions indicates the optimised material properties were not reflective of IVD behaviour. The nucleus pulposus is hyperelastic and poroelastic in nature [28, 29, 30]. Not incorporating these properties into a separate nucleus region may have affected the model IVD pressure predictions. Thus, a more representative material model could result in different IVD pressure optimised material parameter values, and the force‐moment behaviour associated with those pressure optimised values may be more accurate.

The generic material properties of the ligaments and vertebrae may also have affected model accuracy. The longitudinal ligaments were included in the model as they had been left intact during the in vitro testing. While spinal ligament stiffness in general has been shown not to impact model predictions outside of the forces in the ligament itself, anterior longitudinal ligament stiffness may affect the range of motion and IVD pressures during extension [31]. Additionally, the generic ligament properties used were representative of human spinal ligaments instead of porcine, leading to potential inaccuracies. Thus, the generic ligament properties may have had a limited impact on certain axis behaviours. Similarly, homogeneous bone properties can impact spinal segment stiffness for partially modelled vertebrae [32], so the generic bone properties used here may have affected the IVD property optimisations.

Another potential source of model error arises from the pressure transducer. The transducer was present within the IVD during the in vitro testing, but excluded from the model. Thus, any impact the probe may have had on the experimental force‐moment data, due to the stiffer material, would not have been reflected in the model behaviour. As such, the 6‐axis force‐moment optimisation may have been more successful if in vitro pressures had not been collected, removing a source of error between the model and experimental data.

There may also be differences between pressures measured experimentally and predicted by the models. Experimental IVD pressure measurements are not sensitive to precise sensor location within the central nucleus region [33, 34] or type of transducer [35], and there are limited differences between horizontal and vertical transducer orientations in non‐degenerate IVDs [36]. However, a larger diameter transducer may increase IVD pressure measured in species with smaller IVD heights [35]. Porcine IVDs are smaller than species like human or bovine [37], so the pressure measurements may have been affected. Additionally, the model measured pressure as hydrostatic stress rather than the experimental transducer's membrane deformation, and as the nucleus was not modelled explicitly, it may be more sensitive to measurement position. Thus, while trends in IVD pressure would likely be similar, the absolute values could differ, potentially affecting the pressure optimisation accuracy and the optimal material properties.

### 4.2 Impact of Vertebral Alignment

Using model geometry based on the 6‐axis vertebral alignment led to more accurate optimised IVD behaviour in flexion‐extension than model geometry based on the imaged vertebral alignment. Additionally, the vertebral alignment considerably affected the optimal IVD mechanical properties determined, emphasizing the importance of vertebral alignment when optimising using moment data. The flexion‐extension behaviour being optimised may be influenced by both the IVD mechanical properties and the vertebral alignment. Thus, differences in vertebral alignment may result in inaccurate optimised IVD properties that are compensating for the misalignment. Jones and Wilcox [38] previously noted that even direct validation of spinal models can be difficult, as the same overall segment stiffness can be achieved through different combinations of model stiffness components' values. Vertebral alignment appears to be another factor that may influence segment stiffness, and so should be considered when performing direct model optimisation or validation.

To further highlight the importance of vertebral alignment, there was a substantially greater difference in the flexion‐extension stiffness behaviour between the models based on different vertebral alignments (Figure 7), than between the models with different optimisation methods (Figure 6). While there were differences in the optimisation algorithm and parameters optimised, all model versions with geometry based on the imaged vertebral alignment produced similar optimised flexion‐extension behaviour. Thus, the difference in stiffness seems to be due to the vertebral alignment rather than any other variations between the methods.

The improved optimisation when using model geometry based on the in vitro 6‐axis vertebral alignment may have been due to the more accurate representation of in vitro conditions in two respects. Firstly, the vertebral alignment itself affects the way loads are transmitted through the spinal segment. Sagittal curvature of the lumbar spine has been shown to influence spinal forces generated [39]. Thus, it can be expected that differing 6‐axis vertebral alignments would result in differing forces and moments. Secondly, the position and orientation of the model coordinate system, through which kinematics were applied and loads measured, were updated with the vertebral alignment to correspond to the in vitro loading coordinate system. Variations in position of applied loads can impact vertebral stiffness [20], and so ensuring consistency between the experimental and model coordinate systems improves model accuracy. The coordinate systems may not have been in exact agreement, due to the difficulty in precisely defining how the 6‐axis load cell initially aligns with the specimen, resulting in differences in the locations at which forces and moments were determined. However, the improvements in these aspects of the model may have meant there was less compensation for their impact on spinal segment stiffness in the IVD property optimisation.

However, the optimised IVD flexion‐extension behaviour of the model with the 6‐axis vertebral alignment did not completely agree with the experimental behaviour. While the stiffnesses were similar, as observed in the gradients in Figure 7, there was an offset between the model and experimental data. This was likely due to a limitation in the methods used to incorporate the 6‐axis vertebral alignment into the model. During the in vitro testing, as the preload was applied in axial compression, the other five axes were free to float into zero load positions. These five axes of translations and rotations were used to generate the model geometry, to ensure the model stiffness matrix tests began in the same vertebral alignment as the in vitro testing. A two‐stage approach was used to separate kinematics applied to the model that should not generate loading from the preload, which should generate an axial compressive load. However, to maintain the model vertebral alignment as the preload was applied, the other axes were constrained from moving. This resulted in loads being generated in the model in those other five axes, which were not present in the experimental data. An alternative approach could be to allow the model to float in the other axes during the preload. However, there would be no guarantee that the vertebra alignment would be consistent with the in vitro tests. Additionally, some of the vertebral alignment could be accounted for by imaging the specimen after it has been potted and allowed to settle into the neutral position from the in vitro tests prior to applying the preload. However, this may not be feasible depending on imaging and testing facilities.

This assessment was performed for a single porcine specimen, so the results may not generalise to other specimens or species with differing geometry or IVD degeneration. Similarities have been shown in range of motion and neutral zone stiffness between porcine and human lumbar spines, after removal of the posterior elements [40]. Additionally, aging and degeneration of a porcine IVD is similar to a human IVD [41]. The porcine specimen used in this study was likely to be from a Gloucester Old Spot pig in the age range 8–12 months, and the L1‐L2 level tested appeared to be healthy and undamaged. While the exact breed and age of the specimen tested may differ from previous studies, it was deemed appropriately representative of a healthy human lumbar spine for this analysis. However, there are differences between porcine and human lumbar IVDs, for example in IVD height [42], so further investigation is required to confirm these findings in human specimens.

There were other limitations to this investigation of vertebral alignment. The models were only optimised in flexion‐extension, and so the optimised IVD properties determined may not accurately capture stiffness behaviour in the other axes. It is also unknown whether the considerable difference observed between the models with differing vertebral alignments would be present if optimising in another axis. The limitations of the material model used also apply to the vertebral alignment analysis, and so a more representative model may produce differing results. Finally, it should be noted that the removal of the posterior elements between imaging and testing may have influenced the vertebral alignment, so the variation in alignment may not be so pronounced with the posterior elements left intact. Nonetheless, the results from this study indicate that vertebral alignment in modelling is a factor that requires more consideration.

## 5 Conclusions

For this porcine specimen, optimising subject‐specific IVD properties using 6‐axis forces and moments led to more accurate 6‐axis load–displacement behaviour, but less accurate IVD pressures than when optimising with IVD pressures. However, neither optimisation process fully captured spinal behaviours, particularly with regard to the forces and moments. This novel application of 6‐axis force‐moment data for the direct optimisation of IVD properties shows some promise. However, limitations such as the use of a linear orthotropic material model must be addressed to improve the process.

Vertebral alignment was found to have a considerable impact on IVD property optimisation using flexion‐extension data, though it has previously been unexplored in the optimisation of spine models. There was lower error and vastly different optimised IVD properties and stiffness behaviour when model geometry was based on 6‐axis testing vertebral alignment instead of imaged vertebral alignment. While this data only represented a single porcine specimen, it highlights the importance of considering vertebral alignment and the corresponding loading coordinate system orientation during spine model development based on in vitro data.

## Ethics Statement

Ethical approval was not required as no human testing was performed, and animal specimens were acquired through the food chain.

## Conflicts of Interest

The authors declare no conflicts of interest.

## Data Availability

Upon acceptance for publication, Data Files will be deposited to figshare, by Wiley, on behalf of the authors. A CC‐Zero (no rights reserved) license will be applied by default. The data will be assigned a single DOI and will be automatically and permanently associated with the HTML version of the published manuscript.
